# Eosinophil-derived CCL-6 impairs hematopoietic stem cell homeostasis

**DOI:** 10.1038/cr.2018.2

**Published:** 2018-01-12

**Authors:** Chao Zhang, Weiwei Yi, Fei Li, Xufei Du, Hu Wang, Ping Wu, Chao Peng, Man Luo, Wen Hua, Catherine CL Wong, James J Lee, Wen Li, Zhihua Chen, Songmin Ying, Zhenyu Ju, Huahao Shen

**Affiliations:** 1 Key Laboratory of Respiratory Disease of Zhejiang Province, Department of Respiratory and Critical Care Medicine, Second Affiliated Hospital of Zhejiang University School of Medicine, Institute of Respiratory Diseases, Hangzhou, Zhejiang 310009, China; 2 Institute of Aging Research, Leibniz Link Partner Group on Stem Cell Aging, Hangzhou Normal University School of Medicine, Hangzhou, Zhejiang 310036, China; 3 Key Laboratory of Regenerative Medicine of Ministry of Education, Institute of Aging and Regenerative Medicine, Jinan University, Guangzhou, Guangdong 510632, China; 4 National Center for Protein Science Shanghai, Institute of Biochemistry and Cell Biology, Shanghai Institutes for Biological Sciences, Chinese Academy of Sciences, Shanghai 201210, China; 5 Department of Biochemistry and Molecular Biology, Mayo Clinic in Arizona, Scottsdale, Arizona 85259, USA; 6 Department of Pharmacology, Zhejiang University School of Medicine, Hangzhou, Zhejiang 310058, China; 7 Stem Cell Institute, Zhejiang University, Hangzhou, Zhejiang 310058, China; 8 State Key Lab of Respiratory Disease, Guangzhou, Guangdong 510120, China

**Keywords:** eosinophil, CCL-6, hematopoietic stem cell, homeostasis, airway inflammation

## Abstract

Eosinophils (Eos) have been long considered as end-stage effector cells in the hierarchical hematopoietic system. Numerous lines of evidence have suggested that Eos are multifunctional leukocytes with respect to the initiation, propagation and regulation of various inflammatory or immune reactions, especially in allergic diseases. Recent studies have shown that Eos are also required for maintenance of bone marrow plasma cells and differentiation of B cells. However, it remains unclear whether Eos contributes to regulation of hematopoietic stem cell (HSC) homeostasis. Here, we demonstrate that Eos disrupt HSC homeostasis by impairing HSC quiescence and reconstitution ability in wild-type mice following ovalbumin (OVA) challenge and even by causing bone marrow HSC failure and exhaustion in *Cd3δ-Il-5* transgenic mice. The impaired maintenance and function of HSCs were associated with Eos-induced redox imbalance (increased oxidative phosphorylation and decreased anti-oxidants levels). More importantly, using mass spectrometry, we determined that CCL-6 is expressed at a high level under eosinophilia. We demonstrate that CCL-6 is Eos-derived and responsible for the impaired HSC homeostasis. Interestingly, blockage of CCL-6 with a specific neutralizing antibody, restored the reconstitution ability of HSCs while exacerbating eosinophilia airway inflammation in OVA-challenged mice. Thus, our study reveals an unexpected function of Eos/CCL-6 in HSC homeostasis.

## Introduction

Eosinophils (Eos) are mature leukocytes characterized by acidophilic granules in the cytoplasm and Eos-derived granule proteins (EDGPs)^1^. Numerous studies have shown that Eos are multi-functional immune-regulatory cells under various immune and inflammatory processes^2^. Mature Eos are recruited to the inflammatory site by specific chemokines, such as C-C motif chemokine 11 (CCL-11, also known as eosinophil chemotactic protein)^3^, and release an array of cytokines and mediators that act as regulators and effectors. EDGPs, especially major basic protein (MBP), cause tissue damage upon Eos infiltration, for example in asthmatic lung epithelium or during parasitic infections^4^. Eosinophilic airway inflammation during asthma is the classical feature of Eos and targeting Eos represents an effective way to reduce asthma exacerbations.

Eos differentiate from hematopoietic stem cells (HSCs) in the bone marrow (BM) under the regulation of a series of transcription factors (GATA-1, PU.1 and C/EBP)^5^ and cytokines (IL-3, GM-CSF and IL-5)^6^, of which, IL-5 is of prime importance. The *Il-5* gene has been used as a genetic tool to generate mouse strains with altered numbers of Eos to enable in-depth studies of the roles of these cells. Accumulating evidence has suggested new functions of Eos in the regulation of other hematopoietic cells. For example, Eos promote B-cell priming in peripheral blood (PB)^7^ and contribute to the survival of plasma cells in the BM as their niche cells^8^.

Mature blood cells are predominantly short lived; therefore, HSCs are required throughout life to replenish multi-lineage progenitors and their precursors committed to individual hematopoietic lineages. Previous studies have shown that differentiated hematopoietic cells influence HSC homeostasis through feedback mechanisms. Macrophages do so through indirect regulation of osteoblasts and Nestin^+^ perivascular niche cells^9^. Megakaryocytes (MKs) directly serve as niche cells of HSCs to maintain homeostatic quiescence and promote the post-injury regeneration^10^. However, it remains poorly understood how Eos function in the regulation of HSC homeostasis.

In this study, we demonstrate that HSC homeostasis is disrupted both in wild-type (WT) mice challenged with allergic airway inflammation and in *Cd3δ*-*Il-5* transgenic (*Il-5*Tg) mice, where overexpression of IL-5 results in the expansion of the Eos population^11^. The essential role of Eos in regulating HSC homeostasis was confirmed using an eosinophil peroxidase (*Epo*) promoter diphtheria toxin A (DTA)-transgenic (Eos-null) mouse strain^12^. Proteomic analysis of BM supernatant identified an Eos-derived factor, Chemokine (C-C motif) Ligand 6 (CCL-6), which was responsible for the elevated reactive oxygen species (ROS) in HSCs from either *Il-5*Tg or ovalbumin (OVA)-challenged mice, which shared common eosinophilia. Blockage of CCL-6 with a specific neutralizing antibody restored HSC homeostasis both *in vitro* and *in vivo* but aggravated the OVA-induced airway inflammation. This outcome suggests that CCL-6 plays an anti-inflammatory role in allergic airway inflammation but compromises HSC homeostasis. Thus, our data reveal a novel function for Eos in impairing HSC maintenance primarily through the Eos-derived CCL-6.

## Results

### Impaired HSC homeostasis in OVA-induced airway inflammation

To study the function of Eos in HSC homeostasis, we used a chicken OVA-induced asthma model in C57/BL6J WT mice. FACS analysis revealed a significant increase in the levels of Eos (Siglec-F^+^F4/80^+^) in the peripheral blood (PB), BM and spleen (SP) (Supplementary information, Figure S1A). Consistent with previous studies^12^, we found that OVA-mediated airway inflammation and mucus production were dramatically reduced in the absence of Eos (Supplementary information, Figure S1B, S1C and S1D), therefore suggesting a requirement for Eos in the inflammatory response.

**Figure 1:**
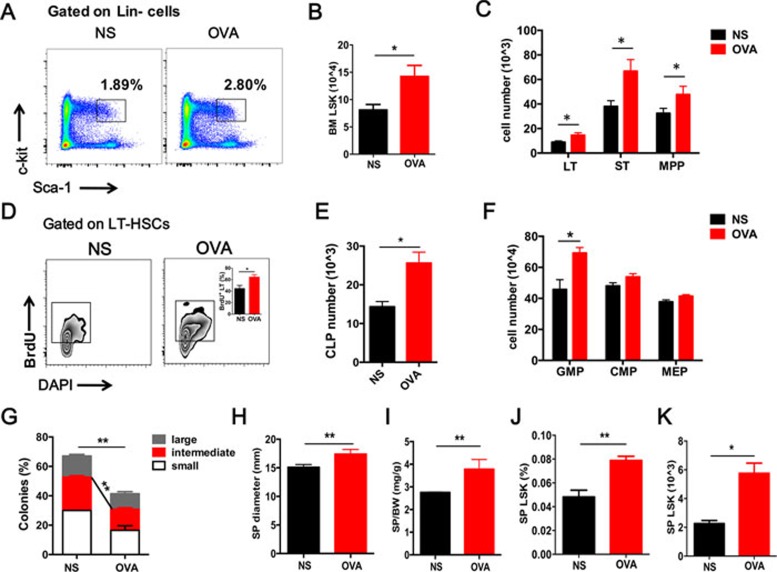
Impaired hematopoietic stem cell homeostasis in allergen-induced airway inflammation. **(A**, **B)** Representative FACS plots and quantification of HSCs (LSKs, Lin^−^Sca-1^+^c-Kit^+^) from the bone marrow (BM) of the OVA-treated mice (OVA) and controls (NS). **(C)** Absolute numbers of stem cells at different stages, including LT-HSCs (CD34^−^Flk2^−^LSKs), ST-HSCs (CD34^+^Flk2^−^LSKs) and MPPs (CD34^+^Flk2^+^LSKs). **(D)** BrdU incorporation assay (24 h) of BM LT-HSCs from the OVA-treated mice (OVA) and controls (NS). **(E**, **F)** Absolute numbers of progenitor cells in the BM. CLP, common lymphoid progenitors; GMP, granulocyte/monocyte lineage progenitors; CMP, common myeloid progenitors; MEP, megakaryocyte/erythroid progenitors. **(G)** A single LT-HSC was sorted into a 96-well plate to perform a colony forming assay. The percentage of colonies was calculated by dividing the original cell number in each colony (*n*= 3). **(H**, **I)** Changes in spleen appearances: long diameter and spleen weight compared with body weight. **(J**, **K)** Percentage of LSK cells in Lin^−^ cells **(J)** and quantification numbers **(K)** in the spleen (SP). Data are shown as the means ± SEM with at least six samples per group. ^*^P < 0.05, ^**^P < 0.01 versus respective controls.

Interestingly, the frequency and absolute number of lineage^−^Sca-1^+^c-Kit^+^ cells (LSKs, FACS analysis procedure are summarized in Supplementary information, Figure S2) in the BM were significantly increased in OVA-treated WT mice (Figure 1A and 1B). Numbers of long-term HSCs (LT-HSCs, CD34^−^Flk2^−^LSKs), short-term HSCs (ST-HSCs, CD34^+^Flk2^−^LSKs) and multi-potential progenitors (MPPs, CD34^+^Flk2^+^LSKs) showed the same tendency (Figure 1C). Further analysis of 5-bromodeoxyuridine (BrdU) incorporation revealed a significantly higher proportion of proliferating cells in HSCs derived from OVA-treated mice compared to normal saline (NS) treated control mice (Figure 1D), suggesting the promotion of HSC proliferation by allergic responses. Further analysis revealed an increase in hematopoietic progenitors and stem cells at different stages of HSC differentiation. Among the progenitors, granulocyte/monocyte lineage progenitors (GMPs) were mainly increased, alongside enhanced Eos differentiation. The numbers of common myeloid progenitors (CMPs), megakaryocyte/erythroid progenitors (MEPs) and common lymphoid progenitors (CLPs) were all increased to some extent (Figure 1E and 1F). To evaluate the *in vitro* function of HSCs from OVA-challenged mice, we performed a single-cell colony units forming assay (CFU) using sorted LT-HSCs from BM. HSCs from OVA-challenged mice formed significantly fewer colonies, especially large and intermediate colonies, compared to WT controls (Figure 1G). The size (long diameter) and weight (ratio with body weight) of the SP were also increased in OVA-treated mice (Figure 1H and 1I), within which an elevated amount of LSKs was detected (Figure 1J and 1K).

The Eos-null mice did not show any obvious difference from their littermates in SP weight, diameter and BM LT-HSCs (Supplementary information, Figure S3A-S3C). However, OVA-treated, Eos-null mice exhibited significantly reduced splenomegaly (Supplementary information, Figure S3D) and attenuated stem and progenitor cell expansion in the BM and the SP compared with their littermates (Supplementary information, Figure S3E-3H), suggesting that Eos-deficiency significantly rescued the disrupted HSC homeostasis. These data together suggested an Eos-dependent disruption of HSC homeostasis in OVA-induced airway inflammation.

### HSC impairment in *Il-5* Tg mice is Eos-dependent

**Figure 2:**
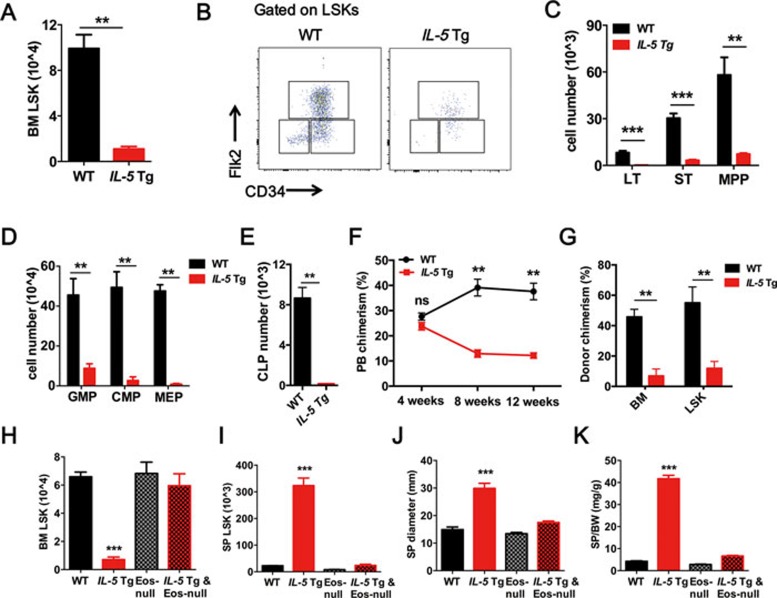
Eos-dependent HSC impairment in *IL-5* Tg mice. **(A)** Quantification of LSKs from the BM of WT and *IL-5* Tg mice. **(B**, **C)** Representative FACS plots and quantification of LT-HSCs, ST-HSCs and MPP-HSCs from the BM. **(D**, **E)** Absolute numbers of progenitor cells in the BM. **(F)** Percentage of donor-derived peripheral blood (PB) cells at 4, 8 and 12 weeks after competitive LSK transplantation. **(G)** Percentage of donor-derived total BM cells and LSKs after 12 weeks of reconstitution in competitive BM transplants. **(H**, **I)** Absolute numbers of LSKs in the BM **(H)** and SP **(I)**. **(J**, **K)** Changes in spleen appearances: long diameter and weight ratio with body weight. Data are shown as the means ± SEM with six samples per group. ^**^P < 0.01, ^***^P < 0.001 versus respective controls. CLP, common lymphoid progenitors; CMP, common myeloid progenitors; GMP, granulocyte/monocyte lineage progenitors; MEP, megakaryocyte/erythroid progenitors.

To investigate the long-term effects of persistent Eos exposure on HSC maintenance and function *in vivo*, we took advantage of the *Il-5* Tg mouse^11^ model in which very high levels of Eos are produced in response to the constitutive T cell-specific overexpression of IL-5. In contrast to the increased number of HSCs observed in WT mice following OVA administration, *Il-5* Tg mice exhibited a significant depletion of HSCs in the BM (Figure 2A) and a dramatic expansion of HSCs in the SP (Supplementary information, Figure S4A). Further analysis revealed a remarkable depletion of LT-HSCs, ST-HSCs, MPPs (Figure 2B and 2C) and hematopoietic progenitors (GMPs; CMPs; MEPs and CLPs) in the BM of these *Il-5* Tg mice (Figure 2D and 2E).

Increased HSC proliferation often results in exhaustion of HSC numbers and a defective reconstitution ability^13,14^. To determine the reconstitution ability of HSCs after exposure to excessive Eos, we performed a competitive BM transplantation experiment^15^ using sorted LSKs (4 000 LSKs plus 1 × 10^6^ competitor BM cells). The results showed that LSKs from *Il-5* Tg mice exhibited a significant defect in reconstitution capacity compared to WT mice (Figure 2F). The percentage of donor-derived BM cells and LSKs was significantly lower in recipient mice transplanted with LSKs from *Il-5* Tg mice than the control group, indicating an exhaustion of HSCs upon transplantation (Figure 2G).

To determine the link between HSC impairment and Eos-mediated activities, we eliminated Eos in *Il-5* Tg mice by crossing them with Eos-null mice^12^. Eosinophil-deficient *Il-5* Tg mice showed a high level of serum IL-5 (Supplementary information, Figure S4B) and a complete absence of Eos in the PB compared to WT mice (Supplementary information, Figure S4C). FACS analysis revealed that the percentage (Supplementary information, Figure S4D, S4E and S4F) and absolute number of LSKs (Figure 2H and 2I) in the BM and SP of double-transgenic mice were similar to those observed in WT mice. Subsequently, we showed that the Eos deficiency rescued the depletion of LT-HSCs, ST-HSCs and MPPs in the BM of *Il-5* Tg mice (Supplementary information, Figure S4G). These data indicate that the HSC defects in *Il-5* Tg mice are dependent on Eos. Similarly, the splenomegaly of *Il-5* Tg mice was also reversed by Eos depletion (Figure 2J and 2K; Supplementary information, Figure S4H). BM cells from WT or *Il-5* Tg mice were transplanted (2 × 10^6^ cells/recipient) into *Il-5* Tg mice or WT littermate controls in a non-competitive transplantation setting. WT donor cells significantly suppressed Eos and restored HSC homeostasis in the BM and SP of *Il-5* Tg-recipient mice, whereas the *Il-5* Tg-donor cells increased the Eos and impaired the HSC homeostasis in the BM and SP of WT recipient mice (Supplementary information, Figure S4I and S4J). Based on the above data, we conclude that the HSC defect associated with *Il-5* overexpression is Eos-dependent.

### HSC impairment is associated with Eos-derived CCL-6-induced redox imbalance

**Figure 3:**
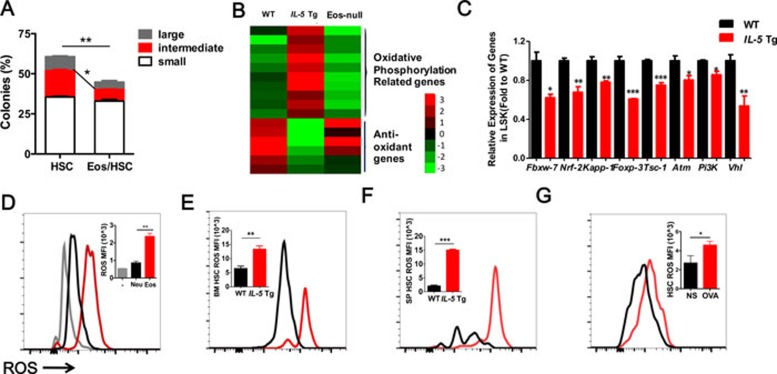
HSC impairment is related to Eos-induced ROS accumulation both *in vitro* and *in vivo*. **(A)** Percentage of large, intermediate and small colonies from a single-colony forming assay of sorted LT-HSCs performed after co-culture with Eos for 2 h. **(B)** Representative RNA-seq analysis heatmap of the oxidative phosphorylation pathway and ROS regulator genes. **(C)** Relative mRNA expression of redox genes in LSKs. **(D)** ROS MFI analysis in sorted LT-HSCs after co-culture with Eos *in vitro* for 2 h. The same numbers of neutrophil are used as control cells. **(E**, **F)** ROS MFI analysis in LT-HSCs from the BM **(E)** and SP **(F)** from *IL-5* Tg mice. **(G)** ROS MFI analysis in sorted LT-HSCs from OVA-challenged mice. Data are shown as the means ± SEM with six samples per group. ^*^P < 0.05, ^**^P < 0.01, ^***^P < 0.001 versus respective controls.

To further analyze the Eos-induced HSC defect, we performed a CFU assay with sorted HSCs (from WT mice) and Eos (from *Il-5* Tg mice), and found that HSCs co-cultured with Eos for 2 h formed fewer large and intermediate-sized colonies (Figure 3A). We then performed a migration assay to measure the migration ability of HSCs in response to SDF-1 (200 ng/ml) for 12 h. This showed that the numbers of HSCs migrating through the trans-membrane were greatly increased when co-cultured with Eos but not with neutrophils (Supplementary information, Figure S5A), suggesting that Eos directly promote HSC mobilization *in vitro*. Next, we sorted HSCs from OVA-treated or *Il-5* Tg mice. The migratory efficiency of HSCs was significantly enhanced in both OVA-treated and *Il-5 Tg* mice compared with the WT controls (Supplementary information, Figure S5B and S5C). Together, these results suggest that Eos are responsible for the HSC impairment both *in vitro* and *in vivo*.

By sorting the LSKs from the WT, *Il-5* Tg and Eos-null mice and performing RNA-seq analysis, we gained insight into the mechanism of Eos-induced HSC defects. The analysis of differential gene expression in LSKs showed that in *Il-5* Tg mice, the oxidative phosphorylation pathway (OXP) was significantly upregulated compared with WT mice (Supplementary information, Figure S5D and S5E). We then analyzed the OXP genes and found that Eos upregulated genes involved in the OXP pathway and downregulated anti-oxidant genes (Figure 3B). This was confirmed by Q-PCR analysis with LSKs sorted from WT and *Il-5* Tg mice (Figure 3C). These data suggest that ROS may be a crucial regulator of the Eos-induced HSC defect. Indeed, several previous studies have shown that extensive ROS production promotes HSC proliferation and mobilization, eventually resulting in exhaustion of functional HSC reserves^16,17,18^, while low ROS are maintained to support HSC long-term repopulation ability. To further substantiate this notion, we performed *in vitro* co-culture experiments of HSCs with either Eos or neutrophils. After 2 h co-culture, we found that Eos, but not neutrophils, directly increased the levels of the intracellular ROS in HSCs (Figure 3D). Similarly, higher levels of intracellular ROS were observed in the BM and SP HSCs of *Il-5* Tg mice (Figure 3E and 3F), likely to result from enhanced mitochondrial superoxide production (Supplementary information, Figure S5F). Similar to our observations in transgenic mice, ROS in HSCs from the OVA-treated group were significantly increased (Figure 3G). Furthermore, the elevated ROS levels in HSCs from *Il-5* Tg mice were also completely rescued both in BM and SP upon Eos depletion (Supplementary information, Figure S5G and S5H). Therefore, we might suggest that the Eos-induced elevation of ROS levels may contribute to the HSC defect.

**Figure 4:**
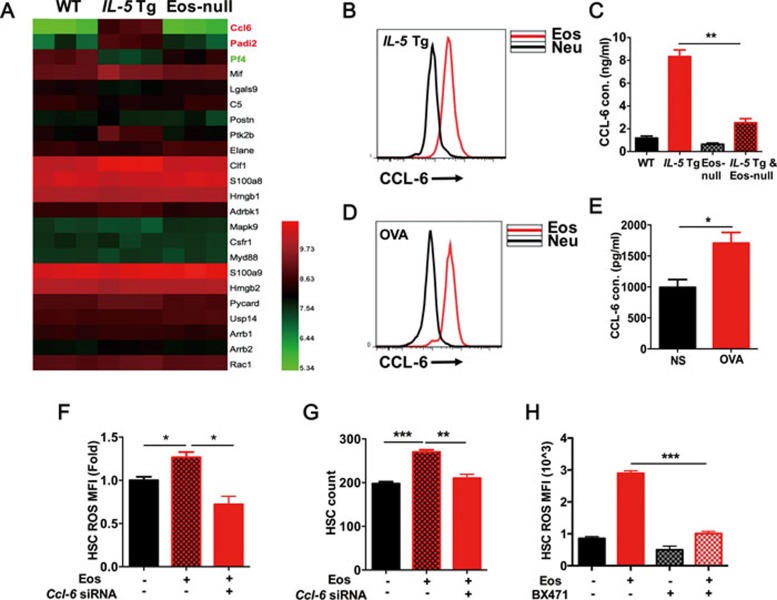
Eos-derived CCL-6 is responsible for disrupted HSC homeostasis. **(A)** Heatmap of screened cytokines and chemokines in bone marrow supernatants of WT, *IL-5* Tg, Eos-null mice analyzed by mass spectroscopy. **(B)** CCL-6 MFI analysis by FACS in Eos and Neu from *IL-5* Tg mice. **(C)** CCL-6 levels determined by ELISA in the serum of WT, *IL-5* Tg, Eos-null and *IL-5* Tg and Eos-null mice. Cons, concentrations. **(D)** CCL-6 MFI analysis by FACS in Eos and Neu from OVA-challenged and control mice. **(E)** CCL-6 levels determined by ELISA in the serum of OVA-challenged and control mice. (**F**) ROS MFI analysis in LT-HSCs co-cultured with Eos transfected with negative control siRNA (NC) or *Ccl-6* siRNA for 2 h. **(G)** HSC migration assay after co-culture for 12 h with Eos transfected with NC or *Ccl-6* siRNA. **(H)** ROS MFI analysis in HSCs co-cultured for 2 h with Eos following treatment with BX471. Data are shown as the means ± SEM with six samples per group. ^*^P < 0.05, ^**^P < 0.01, ^***^P < 0.001 versus respective controls.

To explore the underlying mechanism of Eos-induced redox imbalance in HSCs, a mass spectrometry-based label-free quantitative proteomics strategy was employed to measure cytokine production and secretion from the BM supernatant. From a total of 2 790 identified proteins, 30 proteins were identified as secreted ones in the BM supernatant of the three groups. Among these 30 proteins, 23 cytokines were defined as secreted cytokines. Interestingly, the chemokine (C-C motif) ligand 6 (CCL-6) revealed a significant increase in *Il-5* Tg mice (Figure 4A). This observation was significant because it was previously proposed that Eos-secreted CCL-6 may be related to allergic pathogenesis, although the specific molecular mechanisms are poorly understood^19,20,21^.

FACS analysis of CCL-6 in BM cells of *Il-5* Tg littermates revealed CCL-6 mostly in the SSC^high^ cells (Supplementary information, Figure S6A), which were mainly Eos and neutrophils. However, the mean fluorescence intensity of intracellular CCL-6 staining was remarkably higher in Eos compared to neutrophils in *Il-5* Tg mice (Figure 4B). We then calculated the relative contribution to the increased CCL-6 levels according to the expression levels and the ratio of Eos and neutrophils in these mice. As expected, we found Eos are the main source of CCL-6 in *Il-5* Tg mice (Supplementary information, Figure S6B). By using Q-PCR to analyze *Ccl-6* expression in FACS-sorted Eos and neutrophils from the BM cells of *Il-5* Tg mice, we found that Eos expressed higher level of CCL-6 mRNA (Supplementary information, Figure S6C). This confirms that the CCL-6 in the BM supernatant is secreted from Eos and it is consistent with the higher numbers of Eos in *Il-5* Tg mice. We then measured the serum CCL-6 levels from WT, *Il-5* Tg, Eos-null and *Il-5* Tg & Eos-null mice. *Il-5* Tg mice exhibited very high levels of CCL-6, which were mostly rescued by Eos depletion (Figure 4C). Similarly, we examined the CCL-6 expression in OVA-treated mice and found that CCL-6 levels were elevated upon OVA treatment, in which the Eos expressed significantly higher levels of CCL-6 than neutrophils (Figure 4D and 4E). In the bronchoalveolar lavage fluid (BALF), the relative contribution to CCL-6 was largely from the increased Eos (Supplementary information, Figure S6D), while in the PB and BM, the major cause of the elevated CCL-6 after OVA treatment was mainly attributed to increased Eos but not to neutrophils (Supplementary information, Figure S6E and S6F).

To determine whether CCL-6 from Eos could directly affect HSCs, we performed co-culture experiments. We designed a *Ccl-6* siRNA to transiently deplete CCL-6 in Eos prior to co-culture with LT-HSCs. We were able to transfect ∼50% of Eos in these studies (Supplementary information, Figure S6G), with 98% viability (Supplementary information, Figure S6H) and a significant knockdown efficiency (Supplementary information, Figure S6I). Notably, knockdown of CCL-6 in Eos reduced the elevated levels of ROS in HSCs (Figure 4F) and restored the expansion of HSCs induced by co-culture with Eos (Figure 4G). Furthermore, we used a specific inhibitor (BX471) against CCR1, a putative CCL-6 receptor^20^, to block the effect of CCL-6 on HSCs. BX471 treatment blocked the Eos-induced ROS accumulation in HSCs (Figure 4H) in agreement with previous data. Collectively, these results suggest that CCL-6 plays a key role in the Eos-induced HSC defect.

### Eos-derived CCL-6 impairs HSC homeostasis in OVA-challenged mice

**Figure 5:**
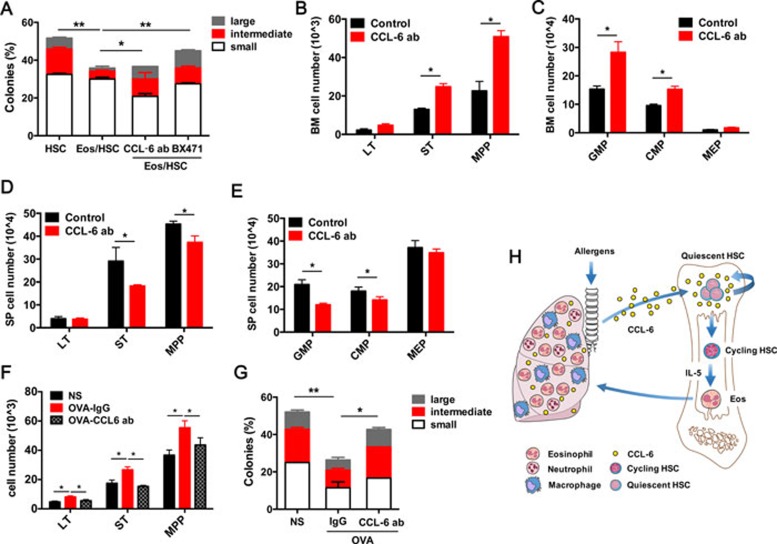
Neutralizing of CCL-6 in *IL-5* Tg and OVA-treated mice rescue HSC impairment. **(A)** Percentage of large, intermediate and small colonies in the single-colony forming assay of sorted LT-HSCs co-cultured with Eos and intervened with the CCL-6 antibody and BX471 (*n* = 3). **(B**, **C)** Absolute numbers of stem cells **(B)** and progenitor cells **(C)** in the BM of *IL-5* Tg and control mice treated with CCL-6-neutralizing antibody. **(D**, **E)** Absolute numbers of stem cells **(D)** and progenitor cells **(E)** in the SP of *IL-5* Tg and control mice treated with CCL-6 antibody. **(F)** Absolute numbers of stem cells in the BM of OVA-challenged mice treated with CCL-6-neutralizing antibody. **(G)** Percentage of large, intermediate and small colonies in a single-colony forming assay performed on LT-HSCs sorted from OVA-challenged mice treated with CCL-6-neutralizing antibody. **(H)** Schematic representation of the roles of Eos and Eos-secreted CCL-6 in regulating HSC function in allergen-induced airway inflammation. Data are shown as the means ± SEM with six samples per group. ^*^P < 0.05, ^**^P < 0.01 versus respective controls.

To verify the function of CCL-6 in the Eos-induced HSC defect, we performed a CFU assay using LT-HSCs co-cultured with Eos in the presence or absence of a CCL-6-neutralizing antibody (5 μg/ml) or BX471 (200 μM). The addition of either neutralizing antibody or the CCR1 inhibitor rescued the defect of HSCs (i.e., their abilities to form colonies, especially the large colonies) (Figure 5A). To validate this finding *in vivo*, CCL-6-neutralizing antibody was administrated to *Il-5* Tg mice at a dose of 5 μg/mouse daily through intraperitoneal injection (i.p.), with the same isotype of Rat IgG_2B_ used as a negative control. After 1-week treatment, we analyzed HSCs in these mice and found that the stem and progenitor cells were significantly restored in the BM (Figure 5B and 5C) and suppressed in the SP (Figure 5D and 5E). This suggests that blocking CCL-6 is able to rescue HSC homeostasis in *Il-5* Tg mice.

We then treated the OVA-challenged mice with the CCL-6-neutralizing antibody (0.2 mg/kg) or its isotype control by intratracheal instillation (i.t.). CCL-6 antibody treatment significantly restored the numbers of LT-HSC (HSCs), ST-HSCs and MPP-HSCs numbers in the BM (Figure 5F). Moreover, we sorted LT-HSCs to perform the CFU assay and found that blocking CCL-6 significantly rescued the colony-forming ability of LT-HSCs from OVA-challenged mice (Figure 5G). These data demonstrate that blocking CCL-6 may effectively reduce the Eos-induced impairment of HSC homeostasis and function.

We also wanted to assess the pathological roles of the above-mentioned activities in airway inflammation. Treatment with the CCL-6-neutralizing antibody increased the cell numbers in the BALF (Supplementary information, Figure S7A). In particular, the numbers of macrophages and Eos were drastically elevated (Supplementary information, Figure S7B). In addition to the effects upon inflammatory cell numbers, Th2-related asthmatic IL-33 levels in the lung homogenates also displayed significant changes (Supplementary information, Figure S7C). An examination of lung pathology in hematoxylin/eosin (H&E) stained sections displayed much higher levels of inflammatory cell infiltration in OVA-challenged mice with the CCL-6 antibody (Supplementary information, Figure S7D). The PAS (periodic acid-Schiff's) staining also showed an elevated mucus secretion in the CCL-6 antibody-treated mice (Supplementary information, Figure S7F). Similarly, quantitation of H&E (Supplementary information, Figure S7E) and PAS (Supplementary information, Figure S7G) staining showed that CCL-6 antibody treatment exacerbated lung inflammation in OVA-challenged mice. On this basis, we can suggest that Eos-derived CCL-6 plays an essential role in suppressing airway inflammation.

## Discussion

In this study, we identified a previously unknown role for Eos in HSC homeostasis. Using mouse models with *Il-5* Tg and Eos-null mouse models, we demonstrated that high doses of CCL-6 secreted from Eos impair the redox homeostasis of HSCs and substantially contribute to HSC exhaustion. CCL-6 has previously been reported to play a role in pulmonary inflammation^20,21^, including disorders in fibrosis, remodeling^22^ and myeloid cell differentiation, and it is chemotactic for macrophages, B cells, CD4^+^ lymphocytes and Eos^23^. In addition, mice deficient in CCR1, the putative CCL-6 receptor^20^ defective mice, have also been reported to exhibit impaired hematopoiesis^24^. We detected a high level of CCL-6 in the serum of *Il-5* Tg mice, most of which were expressed in the Eos. Further experiments revealed that recombinant CCL-6 directly promoted the accumulation of intracellular ROS. RNA-seq analysis of HSCs from *Il-5* Tg mice showed upregulation of oxidative phosphorylation-related genes and downregulation of anti-oxidant genes. Inhibition of CCL-6 decreased the ROS levels and rescued the Eos-induced HSC defects. Interestingly, OVA-challenged mice displayed much worse airway inflammation following treatment with a CCL-6-neutralizing antibody, suggesting that CCL-6 functions as a suppressive mechanism in the asthmatic airway inflammation. Our findings provide the first experimental evidence for a novel mechanistic link between Eos/CCL-6, HSC homeostasis and airway inflammation.

The proper regulation of redox homeostasis in HSCs is critical for their long-term maintenance. In quiescent HSCs, ROS levels are very low. Elevated ROS levels drive HSC differentiation and promote exhaustion of the stem cell pool in the BM, leading to impaired repopulation capacity^16^. Our present work adds a new layer of understanding to a previously unknown function of the Eos-CCL-6-ROS axis in the regulation of HSCs function and could constitute a therapeutic target in several hematopoietic disorders.

In the complex structure of the BM, HSCs are regulated by their adjacent cells in the niche and by the cytokines, chemokines and additional lipid effectors that they produce. In recent years, pro-inflammatory and inflammatory factors such as TNFα, IFNα and IFNγ, have been reported to be related to the emergence and activation of HSCs^25,26,27^. Our present work reinforces the idea that inflammation may be another crucial aspect in the feedback of HSC functions. Equally important, our data suggest an anti-inflammatory role for Eos-derived CCL-6 in the pathophysiology of airway inflammation. Together, our model suggests a complex feedback machinery to control the inflammatory responses, whereby: (1) upon activation Eos secrete CCL-6 to promote HSC proliferation and migration, and subsequently fuel the inflammatory response by increasing the cell reservoirs; (2) in the longer term, CCL-6-mediated HSC proliferation and migration could exhaust the functionality of LT-HSCs, and thereby resulting in compromised immuno-surveillance (Figure 5H); (3) CCL-6 could be an auto-regulatory mediator produced by Eos to inhibit eosinophilic inflammation in eosinophilic disorders such as asthma. Thus, our current findings provide several new insights regarding the expanding roles of Eos and eosinophilia, and is also relevant to the clinical treatment of allergic diseases.

## Materials and Methods

### Mouse strains

C57BL/6 mice (B6-CD45.2) and CD45 locus (B6-CD45.1) mice were purchased from Shanghai SLAC Laboratory Animal Co., Ltd. (Shanghai, China). *Cd3δ* -promoter *Il-5* transgenic (*Il-5* Tg) and *Epo*-promoter DTA transgenic (Eos-null) mice were a gift from Prof James J Lee (Department of Biochemistry and Molecular Biology, Mayo Clinic, USA). *Il-5* Tg and Eos-null mice double-transgenic mice were generated by crossing *Il-5* Tg and Eos-null mice that had been backcrossed to C57BL/6 mice purchased from Shanghai SLSC Laboratory Animal Co., Ltd. (Shanghai, China) for over 20 generations and maintained in a specific pathogen-free facility at the animal center. The mice used for the experiments were 8-12 weeks of age. The genotypes of transgenic mice and their WT littermates were confirmed by PCR analysis of tail snip DNA. All the animal experiments were strictly conducted in accordance with the protocols approved by the Ethics Committee for Animal Studies at Zhejiang University, China.

### OVA-sensitized and challenged asthma model and assessments of airway inflammation^28^

The HSC population was analyzed using a chicken OVA sensitization and challenged asthma model as previously described. Briefly, asthmatic mice were sensitized by an i.p. injection of 20 μg OVA (Sigma-Aldrich, USA) emulsified in Imject Alum (2.25 mg) (Pierce, Rockfield, IL) in a total volume of 200 μL on days 0 and 14. Control mice were injected with the same volume of NS instead. On days 24-28, sensitized mice were subsequently challenged with an aerosol generated from 1.5% OVA in saline and the control mice were challenged with NS alone for 30 min by an ultrasonic atomizer (DeVilbiss, Somerset, PA). At 24 h after the last challenge, mice were killed for analysis. Inflammatory cells from 1 mL of BALF with PBS from the right lung were used to assess inflammation. To identify the cell type and stage, centrifuged cells were stained using Wright-Giemsa buffer according to the manufacturer's instructions. Lung sections from formalin-fixed/paraffin-embedded tissue were additionally stained for the presence of mucin with H&E and PAS reagent to assess inflammation and mucus accumulation. H&E and PAS-staining sections were assigned a score on an arbitrary scale of 0-4 as previously described for the inflammatory situation^29^.

### Flow cytometry analysis of stem cells^30^, eosinophil, neutrophil and intracellular CCL-6

Mouse femurs and tibias were dissected free of muscle and tendons and crushed in PBS (0.2% BSA, pH 7.4) using a mortar and pestle. SPs were isolated, weighed on a microbalance and measured along their major axes with a Vernier caliper. Next, SPs were cut into pieces and minced through a 45 μm mesh (Corning BD Falcon, USA) to yield single-cell suspensions. Cells were counted with a cell counter (Bio-Rad, USA) and adjusted to 1 × 10^8^ cells/mL; 100 μL was taken for FACS analysis and 50 μLwas used for ROS detection. For LSK/HSC staining, cells were first stained with biotin-conjugated anti-mouse lineage cocktail antibodies (Ter-119 (TER-119), Gr-1 (RB6-8C5), CD11b (M1/70), B220 (RA3-6B2), CD4 (RM4-5) and CD8 (53–6.7), Biolegend, USA) for labeling lineage-negative (Lin^−^) cells and then stained with the following antibodies protected from light: CD45.1 (A20, BD), CD45.2 (104, BD), c-Kit (ACK2, eBioscience), Sca-1 (El3–161.7, Biolegend), CD34 (RAM34, BD), Flk2 (A2F10, BD), CD16/32 (93, BD) and IL-7R (A7R34, BD). DAPI was used to exclude dead cells. LSKs were identified as Lin^−^Sca-1^+^c-Kit^+^ cells. LT-HSCs were identified as CD34Flk2^−^LSK cells. For Eos staining, cells were stained with PE-conjugated Siglec-F (E50-2440, BD) and PE-Cy7-conjugated F4/80 (BM8, Biolegend) antibodies. Eos were defined as Siglec-F^+^F4/80^+^ cells. For CCL-6 staining, cells were fixed and permeabilized beforehand with a fix/perm kit (BD) according to the manufacturer's instructions, anti-CCL6 antibody was added before the staining of surface markers for Eos and Neu. Data acquisition was performed using a BD LSRII/Fortessa analyzer. Data were analyzed using FlowJo software.

### Analysis of cell proliferation of LT-HSCs in OVA-treated and control mice

Cell cycle analysis of LT-HSCs (CD34^−^Flk2^−^LSKs) was performed by BrdU staining. For the BrdU incorporation assay, 100 μL BrdU (10 mg/mL, BD Biosciences) per mouse was injected (i.p.) 24 h before killing the OVA-challenged and control mice, followed by administration of 1 mg/mL BrdU in the drinking water. BrdU incorporation was assessed by FACS analysis using a BrdU Flow Kit (BD Biosciences).

### Single-cell colony forming assay

LT-HSCs were sorted into 96-well plates (one cell-one well), and then cultured for 14 days in liquid medium supplemented with 10% FBS, 20% BIT 9500 (StemCell Technologies), 2 mM ℒ-glutamine (Life Technologies), 5 × 10^−5^ M β-ME (Sigma-Aldrich), 10 ng/mL stem cell factor (SCF, Pepro Tech), 10 ng/mL thrombopoietin (TPO, Pepro Tech), 10 ng/mL Interleukin 13 (IL-13, Pepro Tech) and 100 U/mL penicillin/streptomycin. Three classes of colonies were defined under the microscope: large (consisting of more than 10 000 cells); intermediate (1 000-10 000 cells); and small (1-1 000 cells).

### Preparation of blood and serum samples

Mice were lethally anesthetized using an overdose of 1.5% pentobarbital sodium. PB was collected from the right ventricle with a 1-mL sterile syringe. A volume of 200 μL of blood was placed in a heparinized microtube and tested on an automatic blood cell analyzer (Sysmex, Japan) to obtain the total and classified leukocyte cell counts. The remainder of the blood sample was clotted for 2 h at room temperature before centrifuging for 20 min at 2 000× *g*, at 4 °C. The supernatant serum was collected to assay immediately or stored at −80 °C to avoid repeated freeze-thaw cycles.

### Analysis of intracellular ROS levels

Intracellular ROS analysis was performed using an oxidant-sensitive probe, DCFH-DA (Biyuntian, China). A total of 5 × 10^6^ BM cells were loaded into flow tubes for the ROS detection. After staining with LSK antibodies, the cells were incubated with DCFH-DA in a water bath at 37 °C for 30 min. Fluorescence intensity was analyzed on a BD LSR II/Fortessa cell analyzer. Recombinant CCL-6 was purchased from Pepero Tech (Pepero Tech, USA). BX471 was purchased from MedChem Express (MCE, USA).

### c-Kit^+^ cells enrichment and HSC sorting

BM cells were incubated with an APC-conjugated anti-c-Kit antibody followed by anti-APC microbeads (Miltenyi Biotech, Germany). Cells were washed and re-suspended in MACS buffer to a density of 1 × 10^8^/mL and separated through a magnetic large size (LS) column on a magnetic separation plane (Miltenyi Biotech, Germany). Cells remaining on the column were collected for LSK/HSC staining. HSC sorting was performed using a BD Influx cell sorter. Cells were stored at 4 °C before use.

### Bone marrow transplantation assay

Competitive and non-competitive repopulation assays were performed using the CD45.1/CD45.2 congenic systems. For competitive HSC transplantation, 4 000 LSKs sorted from *Il-5* Tg or littermate control (CD45.2) mice mixed with 1 × 10^6^ total BM competitor cells (CD45.1) were transplanted into lethally irradiated (9 Gy) CD45.1/2 recipients intravenously. Chimerism of donor-derived cells in the PB of recipients was analyzed at 4, 8 and 12 weeks after transplantation. After 12 weeks of hematopoietic reconstruction, recipients were killed to determine the donor-derived contribution to the PB and BM. For non-competitive BM transplantation, 2 × 10^6^ total BM cells from donor *Il-5* Tg (CD45.2) or WT (CD45.1) mice of similar age were collected and, respectively, transplanted into WT (CD45.1) and *Il-5* Tg recipients. Recipients were killed after 12 weeks for analysis. The FACS analysis procedure is summarized in the supplemental material (Supplementary information, Figure S2).

### Transwell cell migration assay

Purified HSCs and neutrophils were sorted from WT mice by FACS staining with the above-described methods. Eos were sorted by FACS staining from *Il-5* Tg mice. HSCs were re-suspended in SFEM media (Stem Cell Biology) at a density of 2 × 10^5^/mL. The cell suspension (100 μL) was loaded into the upper chamber of a transwell plate and co-cultured with neutrophils or Eos (10 times the number of HSCs). Next, 600 μL of SFEM media was added to the lower chamber of the transwell plate (pore size, 5 μL, Corning). For the SDF-1 experiment, the controls were set by the presence of additional SDF-1 (200 ng/mL) in the lower chamber. Cells were allowed to migrate at 37 °C for 12 h, and their migration ability was assessed by counting the cell numbers in the lower well.

### Whole transcriptome shotgun sequencing (RNA-seq)

Total mRNA was extracted from the LSKs of WT and *Il-5* Tg mice using the RNeasy Mini Kit (Qiagen). RNA-seq was performed using the Agilent 2200 TapeStation system according to the manufacturer's instructions. The poly-A-containing mRNAs were purified and libraries were built following Illumina TruSeq RNA protocols. Libraries were sequenced using an Illumina HiSeq 2500, and 50 nucleotide-long reads from a single end were obtained.

### Quantitative proteomics

The protein concentration of BM supernatant samples was estimated using a BCA assay kit (Pierce, Rockford, IL). A total of 200 μg proteins of each sample was used for quantification. Protein pellets were first dissolved in 8 M urea, 100 mM Tris-HCl (pH 8.5) and then disulfide bonds were then removed with 5 mM tris-(2-carboxyethyl) phosphine (TCEP). Samples were further alkylated by 10 mM iodoacetamide. The protein mixture was diluted four times and digested with Trypsin at 1:100 (w/w) (Promega). The digested peptides were desalted using a spin column (Pierce), and then analyzed by an in-house packed reversed-phase C18 column (360 μm OD × 75 μm ID) connected to an Easy-nLC 1000 HPLC system by a 3 h-gradient at a flow rate of 300 nL/min. The eluted peptides were ionized and introduced into a Q Exactive mass spectrometry (Thermo, SJ) using a nanospray source. Full MS spectra (from *m/z* 300-1 800) were acquired by the precursor ion scan using the Orbitrap analyzer with resolution *r* = 70 000 at *m/z* 200, followed by 20 MS/MS events in Orbitrap analysis with resolution *r* = 17 500 at *m/z* 200. The 20 most intense ions were sequentially isolated and fragmented under an HCD mode with normalized collisional energy of 27%. Database searching and label-free quantitation was performed by Maxquant (version 1.5.3.30) software against a UniProtKB *Mus musculus* database. The mass tolerances for precursor ions and MS/MS were set at 20 ppm. Trypsin was defined as cleavage enzyme with three most miss cleavage, the mass of the amino acid cysteine was statically modified by +57.02146 Dalton, and the protein FDR was set at 0.01.

### ELISA

Eoxtaxin-2 levels in the BALF and IL-5 levels in the serum were respectively measured using a Mouse IL-5 Quantikine ELISA Kit (R&D Systems Inc.) and a Mouse Eotaxin-2 DuoSet ELISA Kit (R&D Systems Inc.), respectively, according to the manufacturer's instructions. IL-33 levels in the lung homogenates were measured using a Mouse IL-33 Coated ELISA Kit (Thermo Fisher Scientific Inc.). CCL-6 levels in the serum were measured using a Mouse CCL-6/C-C Motif Chemokine 6 ELISA Kit (Sigma-Aldrich, USA) according to the manufacturer's instructions.

### Transient transfection

The *Ccl-6* siRNA transfection in Eos was performed with the transfection reagent following the manufacturer's protocol (SignaGen Laboratories). Briefly, 3 × 10^6^ freshly sorted Eos were placed in 3 mL growth medium for transfection containing 300 μL of transfection regent, including 150 nM siRNA. The siRNA was incubated with Eos for 5 h. The transfection medium was removed and replaced with fresh growth medium (IMDM, 10% FBS) for an additional 7 h. Knockdown efficiency was tested using a Q-PCR procedure. Then, transfected Eos were set up in a co-culture system with LSKs as described above.

### Quantitative-PCR analysis

Total mRNA was extracted from transfected Eos following the manufacturer's instructions. RNA was reverse-transcribed to cDNA with the PrimeScript^™^ RT reagent Kit (TAKARA), and Q-PCR was performed on a StepOnePlue Real-Time PCR system (Applied Biosystems) with SYBR Premix Ex Taq^™^ (TAKARA), all according to the manufacturers' manuals. The mouse primers used for the Q-PCR are as follows:

*Actin*:

forward: 5′-GTCCACCGTGTATGCCTTCT-3′,

reverse: 5′-CTCCTGGTGTCCGAACTGAT-3′

*Ccl-6*:

forward: 5′-AAGAAGATCGTCGCTATAACCCT-3′,

reverse: 5′-GCTTAGGCACCTCTGAACTCTC-3′

*PI3K*:

forward: 5′-CCCACTACTGTAGCCAACAAC-3′,

reverse: 5′-CGTACCAAAAAGGTCCCATCA-3′

*Nrf2*:

forward: 5′-TAGATGACCATGAGTCGCTTGC-3′,

reverse: 5′-GCCAAACTTGCTCCATGTCC-3′

*FoxO3*:

forward: 5′-GCAAGCCGTGTACTGTGGA-3′,

reverse: 5′-CGGGAGCGCGATGTTATCC-3′

*Tsc1*:

forward: 5′-ATGGCCCAGTTAGCCAACATT-3′,

reverse: 5′-CAGAATTGAGGGACTCCTTGAAG-3′

*ATM*:

forward: 5′-CCAGCTTTTTGATGCAGATACCA-3′,

reverse: 5′-CTTCCCAGCCTACGTCTATTTTC-3′

*VHL*:

forward: 5′-CTCAGCCCTACCCGATCTTAC-3′,

reverse: 5′-ACATTGAGGGATGGCACAAAC-3′

*Fbxw-7*:

forward: 5′-TACAAACTGGAGACGAGGAGAA-3′,

reverse: 5′-CCACAAAACTGTAGGCATGTGAT-3′

*Keap1*:

forward: 5′-TCGAAGGCATCCACCCTAAG-3′,

reverse: 5′-CTCGAACCACGCTGTCAATCT-3′

### CCL-6 neutralizing antibody treatment on *Il-5* Tg and OVA-challenged mice

Rat anti-mouse CCL-6-neutralizing antibody (MAB487, R&D) dissolved in PBS was given to *Il-5* Tg mice at a dose of 5 μg/mouse daily i.p. for 1 week. The same isotype IgG2B was used as negative controls. At the 8th day, mice were killed to analyze their stem cell and progenitor cell populations in the BM and SP following FACS. The same CCL-6 antibody was used to treat OVA-challenged mice at a dose of 5 μg/mouse daily 2 h before each OVA challenge. Twenty-four hours after the last OVA challenge mice were killed to analyze BM stem cells by FACS. LT-HSCs were also sorted to perform a single-cell colony forming assay. Airway inflammation was also assessed according to the above-described methods.

### Statistical analysis

Statistical analysis was performed using an unpaired two-tailed Student's *t* test and GraphPad Prism software. Data are shown as the means ± SEM. *P* < 0.05 was considered statistically significant.

## Author Contributions

HS, ZJ and SY initiated the study and developed the concept of the paper. CZ and WY performed all the experiments. FL, ML and WH participated in the animal experiments. PW, CP and CW performed the quantitative proteomics and data analysis. CZ, WY, FL, XD, HW, WL, ZC, SY, ZJ and HS analyzed and interpreted the data. HS, ZJ, SY, CZ and JJL wrote the manuscript.

## Competing Financial Interests

The authors declare no competing financial interests.

## Supplementary Information

Supplementary information, Figure S1 Eos and inflammation analysis in OVA-treated WT and Eos-null mice.

Supplementary information, Figure S2 FACS staining and analysis procedure of HSC cells.

Supplementary information, Figure S3 Eos depletion rescues the HSC defect in *IL-5* Tg mice.

Supplementary information, Figure S4 Eos depletion rescues the HSC defect in *IL-5* Tg mice.

Supplementary information, Figure S5 Eos-induced intracellular ROS accumulation in HSCs is responsible for disrupted HSC homeostasis.

Supplementary information, Figure S6 Eos are the major cause in *Il-5* Tg and OVA-treated mice.

Supplementary information, Figure S7 CCL-6 neutralizing antibody alleviated airway inflammation in OVA-treated mice.
