# Supplementary material for: Eosinophil-derived CCL-6 impairs hematopoietic stem cell homeostasis
Source: Cell Res. 2018 Jan 12;28(3):323–35. doi: 10.1038/cr.2018.2 (PMC5835778; doi:10.1038/cr.2018.2)
Supplement: Supplementary information, Figure S1 — Eos and inflammation analysis in OVA-treated WT and Eos-null mice. [file cr20182x1.pdf]

Supplementary Figure 1: Eos and inflammation analysis in OVA-treated WT and Eos-null mice.

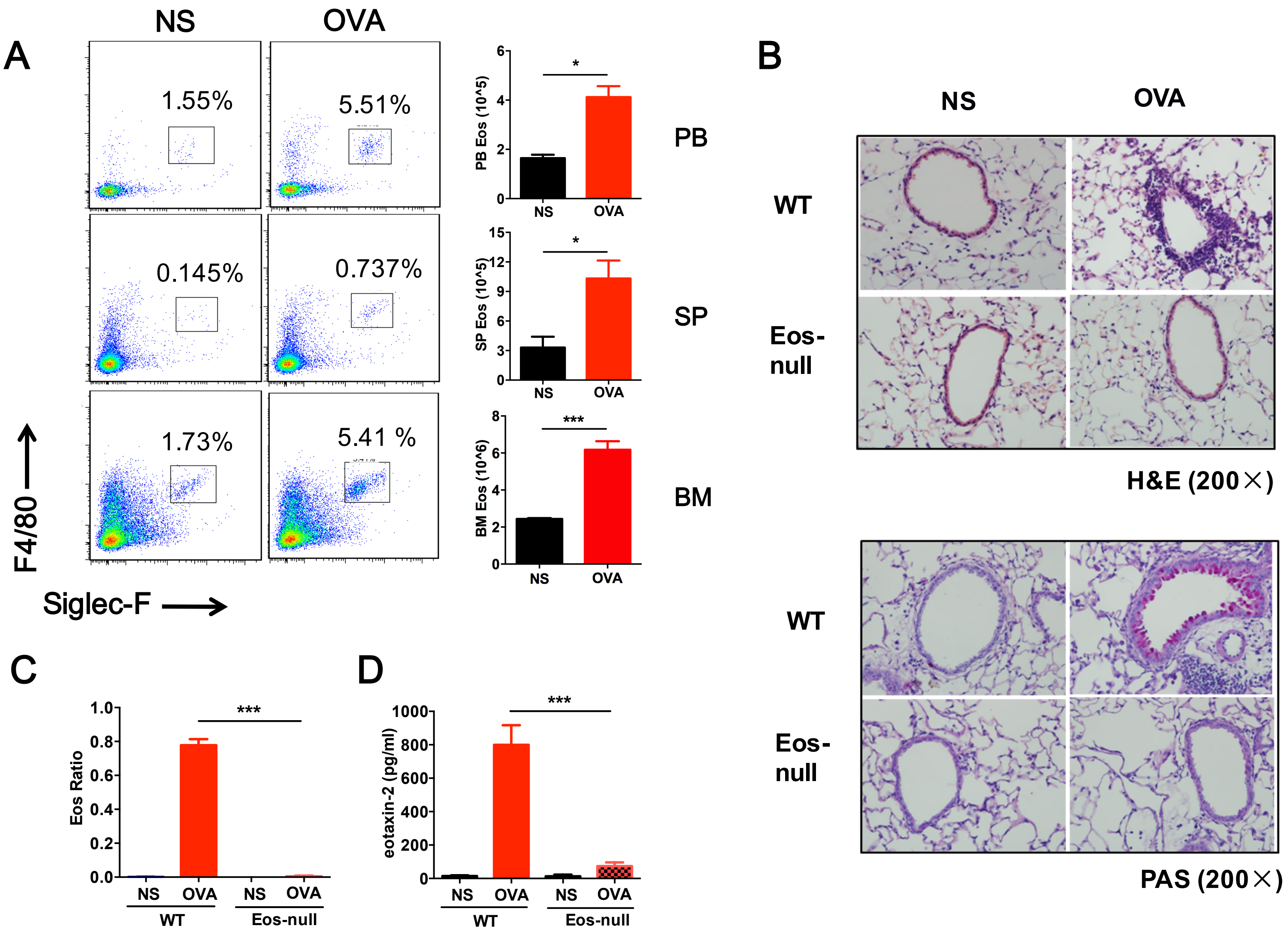

Supplementary Figure S1 Eos and inflammation analysis in OVA-treated WT and Eos-null mice.

(A) Representative FACS plots and quantification of Eos in the bone marrow (BM), spleen (SP), and peripheral blood (PB) of OVA-treated and control mice. **(B)** Representative H&E and PAS staining lung sections observed under microscope with 200 times amplification. **(C)** Percentage of Eos in the bronchoalveolar lavage fluid (BALF) inflammatory cells quantified by Wright-Giemsa staining. **(D)** Eotaxin-2 levels in BALF measured using a specific ELISA. Data are shown as the means ± SEM with 6 samples per group. *$p < 0.05$, ***$p < 0.001$ versus the respective controls.
