# Supplementary figures and images for: Eosinophil-derived CCL-6 impairs hematopoietic stem cell homeostasis
Source: Cell Res. 2018 Jan 12;28(3):323–35. doi: 10.1038/cr.2018.2 (PMC5835778; doi:10.1038/cr.2018.2)

Supplementary Figure S2 FACS staining and analysis procedure of HSC cells.

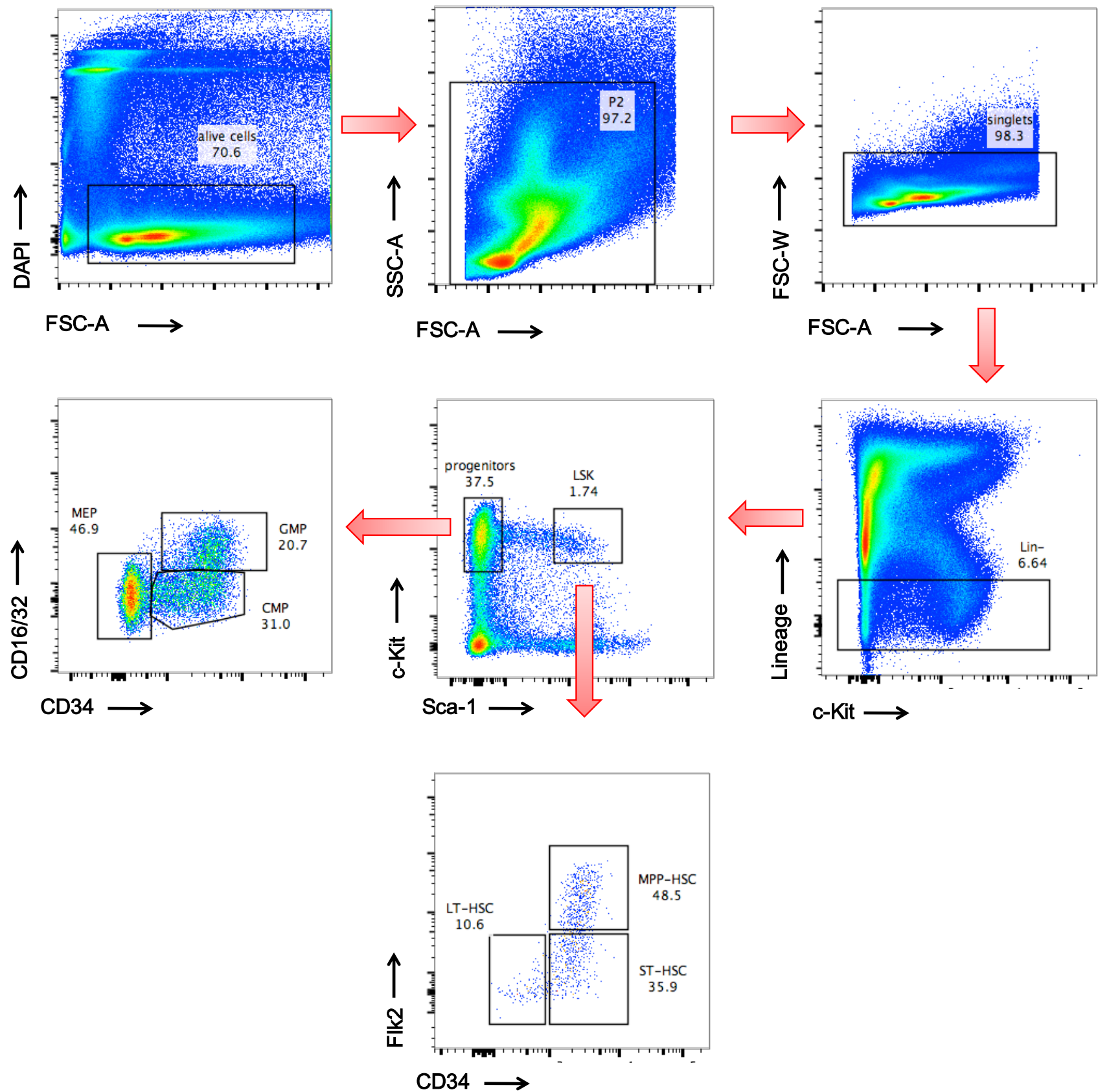

Supplement: Supplementary information, Figure S2 — FACS staining and analysis procedure of HSC cells. [file cr20182x2.pdf]
