# Supplementary material for: Eosinophil-derived CCL-6 impairs hematopoietic stem cell homeostasis
Source: Cell Res. 2018 Jan 12;28(3):323–35. doi: 10.1038/cr.2018.2 (PMC5835778; doi:10.1038/cr.2018.2)
Supplement: Supplementary information, Figure S3 — Eos depletion rescues the HSC defect in IL-5 Tg mice. [file cr20182x3.pdf]

Supplementary Figure 3: Eos depletion rescues the HSC defect in *IL-5* Tg mice.

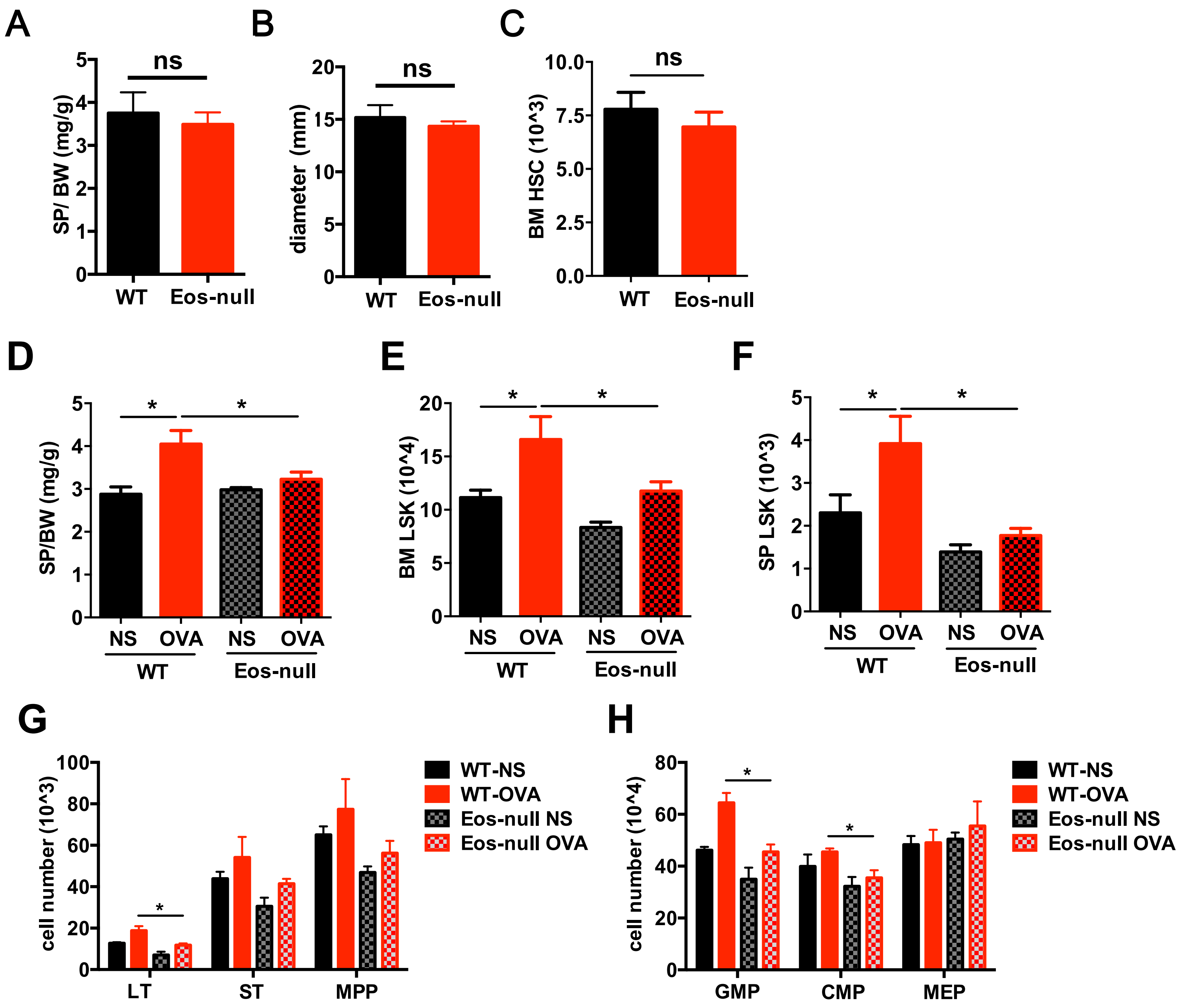

Supplementary Figure S3 Eos depletion rescues the HSC defect in *Il-5* Tg mice.

(A, B) Changes in the spleen appearances: long diameter (A) and spleen weight (B) compared with body weight. **(C)** Absolute total HSC numbers in the BM of Eos-null mice and their littermates. **(D)** Spleen weight compared with body weight of Eos-null mice and their littermates under OVA-treatment. **(E, F)** Absolute total LSK numbers in the BM (E) and SP (F) of Eos-null mice and littermates under OVA-treatment. **(G, H)** Absolute numbers of stem cells (G) and progenitor cells (H) in the BM of Eos-null and littermates under OVA-treatment. Data are shown as the means ± SEM with 6 samples per group. ns: not significant; $^{*}p < 0.05$ versus the respective controls.
