# Supplementary material for: Eosinophil-derived CCL-6 impairs hematopoietic stem cell homeostasis
Source: Cell Res. 2018 Jan 12;28(3):323–35. doi: 10.1038/cr.2018.2 (PMC5835778; doi:10.1038/cr.2018.2)
Supplement: Supplementary information, Figure S4 — Eos depletion rescues the HSC defect in IL-5 Tg mice. [file cr20182x4.pdf]

Supplementary Figure 4: Eos depletion rescues the HSC defect in *IL-5* Tg mice.

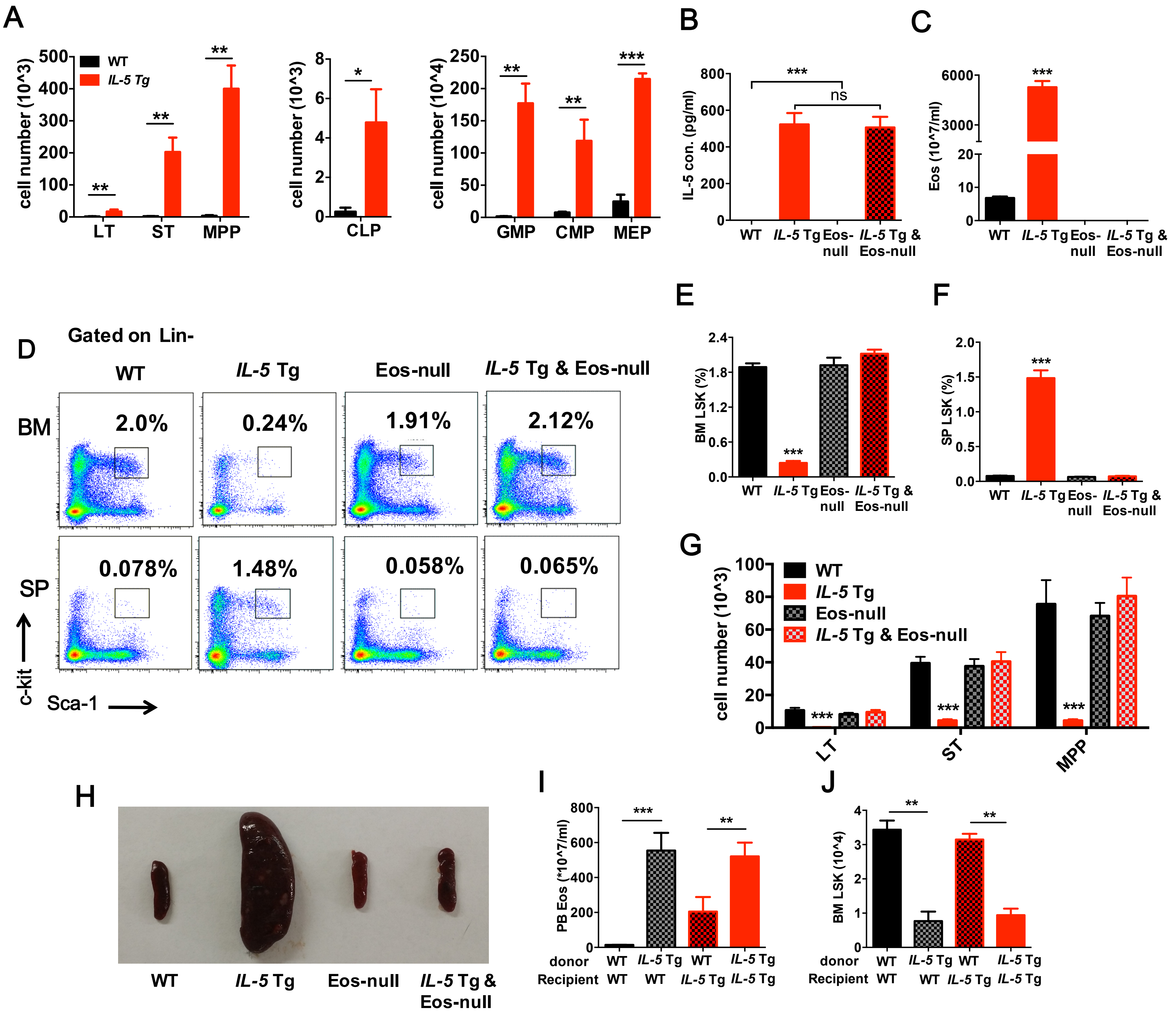

Supplemental Figure S4 Eos depletion rescues the HSC defect in Il-5 Tg mice.

(A) Quantification numbers of stem cells and progenitor cells population by FACS analysis in the SP of Il-5 Tg mice and their littermates. **(B)** IL-5 levels in the serum determined by ELISA in Il-5 Tg mice. **(C)** Eos quantification in the peripheral blood (PB) analyzed by FACS and white blood cell (WBC) count. **(D)** Representative FACS plots of LSKs from the BM and SP in Il-5 Tg mice crossed with Eos-null mice. **(E, F)** Percentage of LSKs in the Lin$^-$ cells in the BM and SP. **(G)** Absolute numbers of stem cells in the BM of Il-5 Tg and Eos-null crossed mice. **(H)** Spleen appearance. **(I, J)** Absolute numbers of Eos in the PB and LSKs in the BM by FACS analysis after a 12-week noncompetitive transplantation. Data are shown as the means ± SEM with at least 6 samples per group. *$p < 0.05$, **$p < 0.01$, ***$p < 0.001$ versus respective controls.
