# Supplementary material for: Eosinophil-derived CCL-6 impairs hematopoietic stem cell homeostasis
Source: Cell Res. 2018 Jan 12;28(3):323–35. doi: 10.1038/cr.2018.2 (PMC5835778; doi:10.1038/cr.2018.2)
Supplement: Supplementary information, Figure S5 — Eos-induced intracellular ROS accumulation in HSCs is responsible for disrupted HSC homeostasis. [file cr20182x5.pdf]

Supplementary Figure 5: Eos-induced intracellular ROS accumulation in HSCs is responsible for disrupted HSC homeostasis.

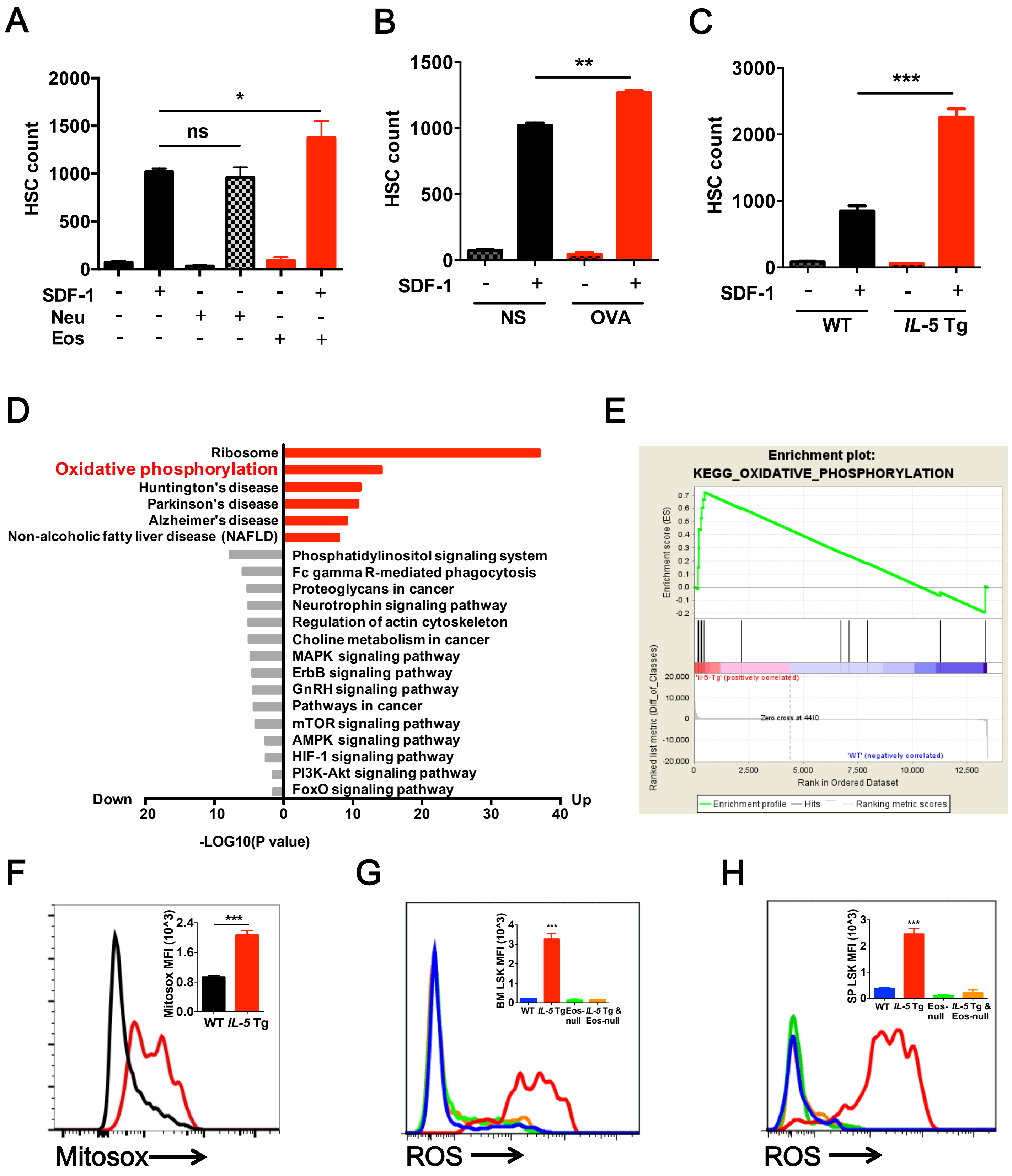

Supplemental Figure S5 Eos-induced intracellular ROS accumulation in HSCs is responsible for disrupted HSC homeostasis.

(A) HSCs sorted from WT mice and cocultured with Neu/Eos or SDF-1 (200 ng/ml). Number of HSCs that had migrated from upper wells was counted after 12 h culture *in vitro*. (**B, C**) HSC migration assay performed with HSCs sorted from the BM of OVA mice and *Il-5* Tg mice following SDF-1 treatment. The number of HSCs that had migrated from upper wells was counted after 12 h culture *in vitro*. **(D, E)** KEGG (D) and GSEA (E) Pathway analysis shows enrichment of the oxidative phosphorylation pathway in *Il-5* Tg versus WT cells (FDR<0.05). **(F)** Mitosox MFI analysis in HSCs from WT and *Il-5* Tg mice. **(G, H)** ROS MFI analysis in the BM and SP HSCs from the double-transgenic mice. Data are shown as the means ± SEM with at least 6 samples per group. ns: not significant, $*p < 0.05$, $**p < 0.01$, $***p < 0.001$ versus respective controls.
