# Supplementary material for: Eosinophil-derived CCL-6 impairs hematopoietic stem cell homeostasis
Source: Cell Res. 2018 Jan 12;28(3):323–35. doi: 10.1038/cr.2018.2 (PMC5835778; doi:10.1038/cr.2018.2)
Supplement: Supplementary information, Figure S6 — Eos are the major cause in Il-5 Tg and OVA-treated mice. [file cr20182x6.pdf]

Supplementary Figure 6: Eos are the major cause in *IL-5* Tg and OVA-treated mice.

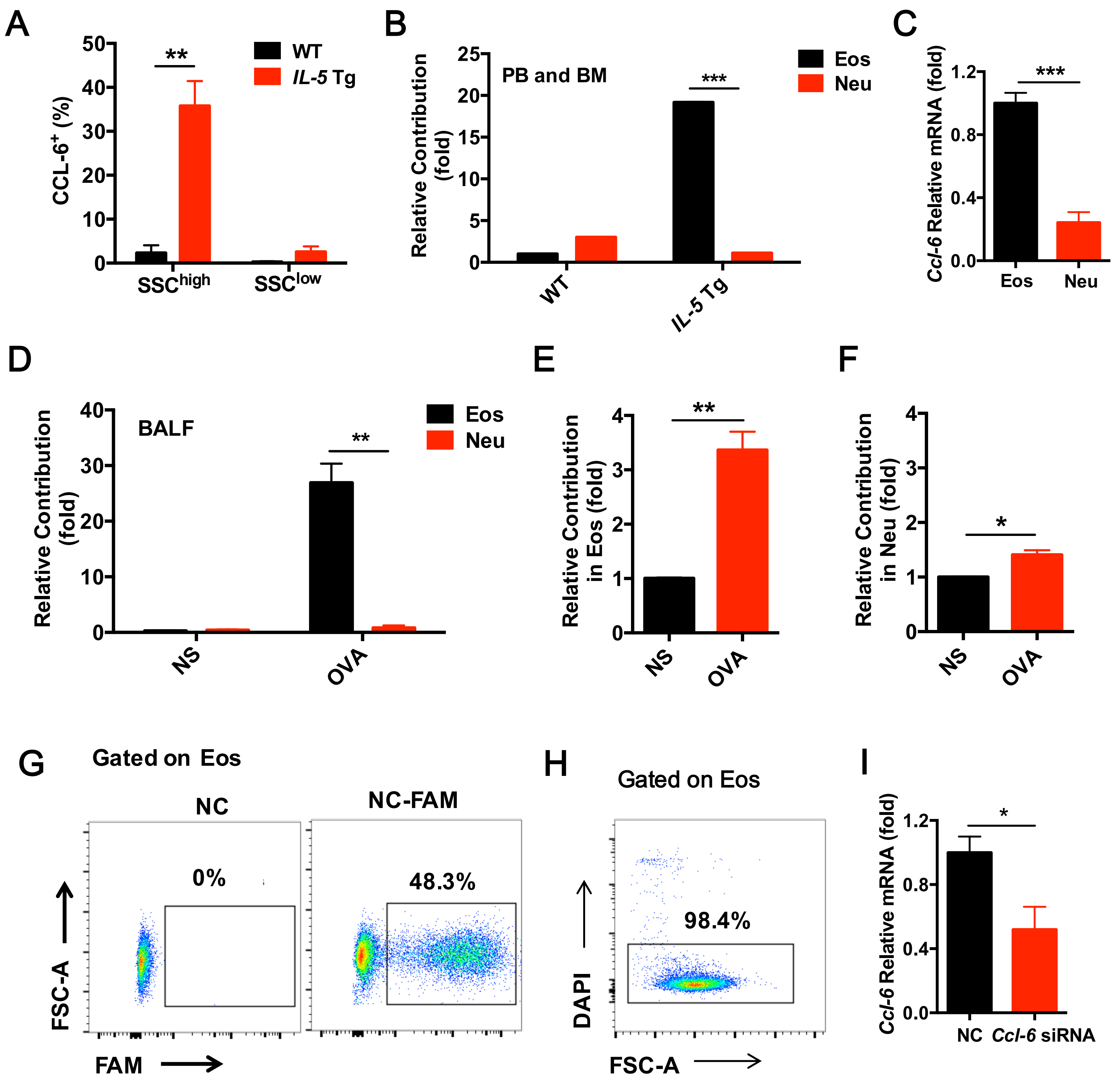

Supplemental Figure S6 Eos are the major cause in *Il-5* Tg and OVA-treated mice. **(A)** Percentage of CCL-6^{+} cells in the BM SSC$_{high}$ and SSC$_{low}$ from WT and *Il-5* Tg mice. **(B)** Relative contribution of Eos and neutrophils on CCL-6 expression from PB and BM in WT and *Il-5* Tg mice. **(C, D)** Relative contribution of Eos and neutrophils on CCL-6 expression from BALF in OVA-treated mice. **(E, F)** CCL-6 relative contribution of Eos and Neu in the BM and PB of OVA-treated mice, respectively. **(G)** Transfected efficiency of Eos performed with negative control siRNA (NC) with FAM fluorescence after 5 h. **(H)** Representative FACS plots of the percentage of surviving Eos after transfection and culture for 12 h. **(I)** *Ccl-6* mRNA expression in transfected Eos following negative control siRNA or *Ccl-6* siRNA transfection. Data are shown as the means ± SEM with at least 4 samples per group. *p < 0.05, **p < 0.01, ***p < 0.001 versus respective controls.
