# Supplementary material for: Eosinophil-derived CCL-6 impairs hematopoietic stem cell homeostasis
Source: Cell Res. 2018 Jan 12;28(3):323–35. doi: 10.1038/cr.2018.2 (PMC5835778; doi:10.1038/cr.2018.2)
Supplement: Supplementary information, Figure S7 — CCL-6 neutralizing antibody alleviated airway inflammation in OVA-treated mice. [file cr20182x7.pdf]

Supplementary Figure 7: CCL-6 neutralizing antibody alleviated airway inflammation in OVA-treated mice.

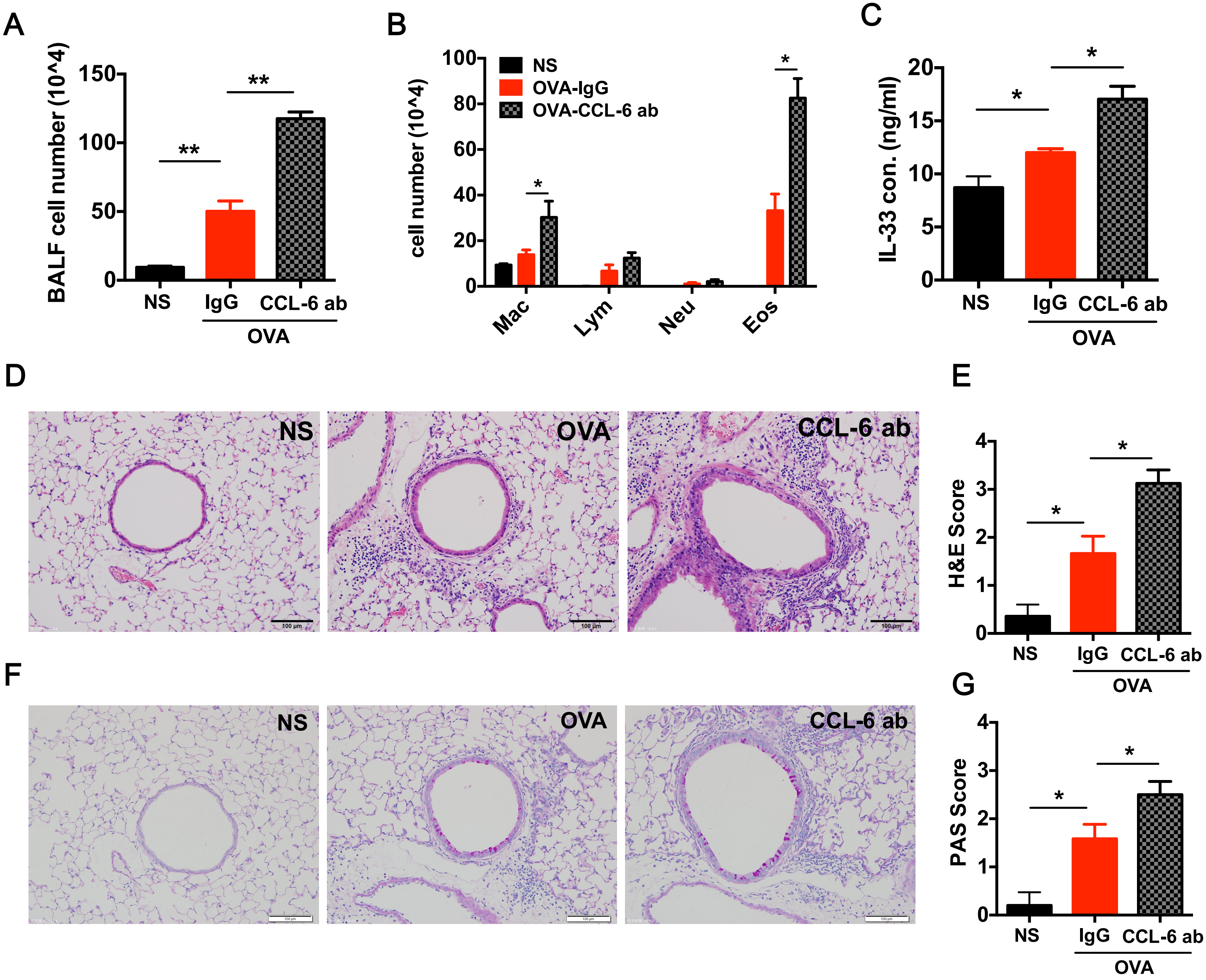

Supplemental Figure S7 CCL-6 neutralizing antibody alleviated airway inflammation in OVA-treated mice.

(A) Total inflammatory cell quantification in the bronchoalveolar lavage fluid (BALF) from CCL-6 neutralizing antibody treat OVA mice (OVA CCL-6 ab), negative IgG control mice (OVA IgG). **(B)** Cell populations and quantification of BALF cells with Wright-Giemsa staining under a microscopy of 400 times amplification. **(C)** IL-33 levels in the lung homogenates determined with ELISA in the CCL-6 antibody-treated and their control mice. **(D, F)** Representative H&E (D) and PAS (F) staining of lung pathological sections (Bars: 100 μm). **(E, G)** Evaluation of scores on lung H&E and PAS sections in a double-blinded test by three individuals. Data are shown as the means ± SEM with at least 6 samples per group. $*p < 0.05$, $**p < 0.01$ versus respective controls.
